# Methylsulfonylmethane Induces p53 Independent Apoptosis in HCT-116 Colon Cancer Cells

**DOI:** 10.3390/ijms17071123

**Published:** 2016-07-15

**Authors:** Arzu Zeynep Karabay, Asli Koc, Tulin Ozkan, Yalda Hekmatshoar, Asuman Sunguroglu, Fugen Aktan, Zeliha Buyukbingol

**Affiliations:** 1 Faculty of Pharmacy, Department of Biochemistry, Ankara University, 06100 Ankara, Turkey; aktanf@ankara.edu.tr (F.A.); zbuyukbingol@ankara.edu.tr (Z.B.); 2 Faculty of Pharmacy, Ankara University, 06100 Ankara, Turkey; 3 Faculty of Medicine, Department of Medical Biology, Ankara University, 06560 Ankara, Turkey; yaldahekmatshoar@yahoo.com (Y.H.); asungur@medicine.ankara.edu.tr (A.S.)

**Keywords:** MSM, HCT-116, apoptosis, Bim, colon cancer, JNK

## Abstract

Methylsulfonylmethane (MSM) is an organic sulfur-containing compound which has been used as a dietary supplement for osteoarthritis. MSM has been shown to reduce oxidative stress and inflammation, as well as exhibit apoptotic or anti-apoptotic effects depending on the cell type or activating stimuli. However, there are still a lot of unknowns about the mechanisms of actions of MSM. In this study, MSM was tested on colon cancer cells. 3-(4,5-Dimethylthiazol-2-yl)-2,5 diphenyltetrazolium bromide (MTT) assay and flow cytometric analysis revealed that MSM inhibited cell viability and increased apoptotic markers in both HCT-116 p53 +/+ and HCT-116 p53 −/− colon cancer cells. Increased poly (ADP-ribose) polymerase (PARP) fragmentation and caspase-3 activity by MSM also supported these findings. MSM also modulated the expression of various apoptosis-related genes and proteins. Moreover, MSM was found to increase c-Jun N-terminal kinases (JNK) phosphorylation in both cell lines, dose-dependently. In conclusion, our results show for the first time that MSM induces apoptosis in HCT-116 colon cancer cells regardless of their p53 status. Since p53 is defective in >50% of tumors, the ability of MSM to induce apoptosis independently of p53 may offer an advantage in anti-tumor therapy. Moreover, the remarkable effect of MSM on Bim, an apoptotic protein, also suggests its potential use as a novel chemotherapeutic agent for Bim-targeted anti-cancer therapies.

## 1. Introduction

Natural products with low toxicity have been gaining attention for the treatment of various cancers, including colorectal carcinoma, which is the third most common cancer in the world [1]. One of these compounds, methylsulfonylmethane (MSM) is an organic dietary supplement which has been marketed for the last 20 years especially to alleviate arthritic symptoms, support joint flexibility, and maintain healthy joints. In animal studies, MSM has been shown to be well tolerated at an acute dose of 2 g/kg and not to cause any maternal or developmental toxicity up to 1000 mg/kg/day. These concentrations are five times higher than the doses used in humans [2,3]. Therefore, the low toxicity index and the probable bio-activity of MSM have prompted researchers to investigate the biological effects of MSM more elaborately. MSM has been shown to exert anti-inflammatory [4], anti-cancer [5], and anti-oxidative [6] effects in different studies. In recent years, researchers have become increasingly interested in the action mechanism of MSM on apoptosis in different in vitro and in vivo cancer models. It was reported that combination therapy of MSM (200 mM) and tamoxifen inhibited breast cancer tumor growth and metastasis both in vitro and in vivo by inhibiting the Janus kinase 2 (JAK2)/signal transducer and activator of transcription 5b (STAT5b) pathway [7]. In another study, suppressive activity of MSM against hepatic tumor development was examined and it was found that MSM decreased the growth of three different liver cancer cell lines by cleaving caspase-3, caspase-8, and poly (ADP-ribose) polymerase (PARP), and slightly decreasing Bcl-2 at 500 mM concentration [8]. MSM has also been reported to repress bladder tumor growth at 300 mM concentration when used in combination with JAK2 inhibitor AG490 by reducing STAT3, STAT5b, IGF-1R, VEGF, and VEGF-R2. These signaling molecules are related to the growth, progression, and metastasis of human bladder cancer [9].

It has been suggested that dysregulation of cell proliferation and apoptotic pathways leads to tumorigenesis, accumulation of cancer cells, resistance to chemotherapeutic drugs, angiogenesis, invasion, and metastasis [10]. Various intracellular signaling pathways regulate apoptosis, while the initiation and execution of this programmed cell death depend on the activation of the receptor- and/or mitochondrial-dependent death pathways [11]. Bcl-2 family proteins regulate the mitochondrial pathway of apoptosis as a response to various cytotoxic signals. The ratio of Bax/Bcl-2 is an important indicator of execution of apoptosis and the balance between the pro-apoptotic (Bax, Bak, Bim, Bid) and anti-apoptotic (Bcl-2, Bcl-xL, Bcl-w) Bcl-2 family proteins determine the fate of cells [12,13]. Recently, the pro-apoptotic molecule Bim has been gaining serious attention as a critical regulator of tissue homeostasis. Its expression level is strictly controlled in both transcriptional and post-transcriptional levels, which depends on cell, tissue, and apoptotic stimuli [14]. Bim has been shown to mediate tumor cell death in response to chemotherapeutic agents and, therefore, Bim-targeting therapies are considered as promising strategies to fight cancer [15].

In addition to Bcl-2 family proteins, members of the p53 family are also important players in the cellular stress response in cancer and, therefore, these genes may serve as therapeutic targets [16]. Apoptosis and cell cycle arrest are two main mechanisms which mediate the response of p53 to DNA damage [17]. Cells lacking the *p53* gene can also undergo apoptosis via the modulation of different proteins. Moreover, several agents have been shown to induce apoptosis in cancer cells with deleted or mutant p53 [18,19,20]. p53 upregulated modulator of apoptosis (PUMA) is another pro-apoptotic protein which is involved in both p53 dependent and independent apoptosis. PUMA can interact with Bcl-2-like proteins, to free Bax and/or Bak, which then transmit apoptotic signals to the mitochondria. [21,22].

In addition to these apoptotic genes and proteins, the apoptotic process is affected by various other signaling pathways, including the mitogen-activated protein kinases (MAPKs) pathway. MAPK family members, including p44/42 (extracellular signal-regulated kinase, ERK1/2), JNK (c-Jun N-terminal kinases), and p38 MAPK are crucial for the regulation of cellular programs, such as proliferation, differentiation, development, transformation, apoptosis, and control of cellular responses to cytokines and stress [23,24]. JNK may exhibit both apoptotic or anti-apoptotic roles and dysregulation of the JNK pathway has been linked to cancer [25,26]. Apoptosis is mediated by activated JNK through a phosphorylation mechanism induced by UV irradiation, heat shock, chemotherapy, pro-inflammatory cytokines, and growth factors [27,28,29]. JNK 1- and JNK 2-deficient mouse embryonic fibroblasts have been shown to exhibit resistance to apoptosis induced by ultraviolet irradiation [30]. Various apoptotic or autophagic stress signals may also stimulate JNK [24]. JNK has been reported to activate or inactivate p53, Bcl-2, and Bcl-xL [31,32,33]. Thus, targeting the JNK pathway is an important strategy in treatment and prevention of cancer.

In this study we aim to elucidate the action mechanisms of MSM on apoptosis in HCT-116 colon cancer cells. The effects of MSM on important regulators of apoptosis, such as Bcl-2 family members, p53, and MAPKs, were examined.

## 2. Results

### 2.1. Methylsulfonylmethane (MSM) Inhibited Proliferation of HCT-116 p53 +/+ and HCT-116 p53 −/− Colon Cancer Cells

**Figure 1:**
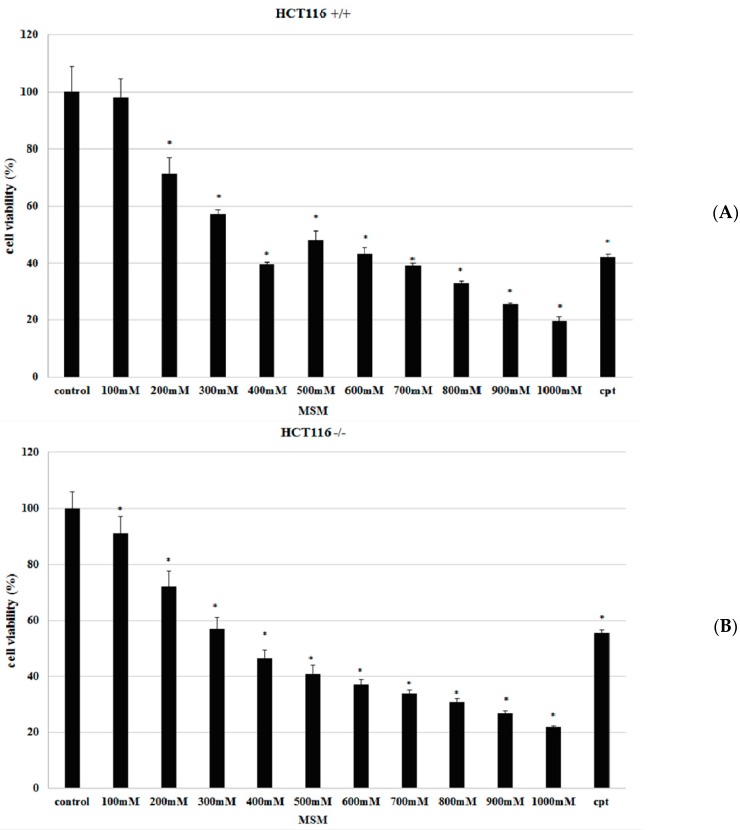
Effect of methylsulfonylmethane (MSM) (100–1000 mM) on cell viability of HCT-116 p53 +/+ and HCT-116 p53 −/− colon cancer cells. HCT-116 p53 +/+ and HCT-116 p53 −/−colon cancer cells were incubated with MSM for 24 h before analyzing viability with 3-(4,5-Dimethylthiazol-2-yl)-2,5 diphenyltetrazolium bromide (MTT) assay. Treatment of HCT-116 p53 +/+ (**A**) and HCT-116 p53 −/− (**B**) colon cancer cells with MSM decreased cell viability. Camptothecin (cpt) (30 µg/mL) was used as a positive control. Data were shown as means ± SD of three independent experiments (* shows significant differences from the control group, *p* < 0.001).

To identify the effects of MSM on proliferation, HCT-116 p53 +/+ and HCT-116 p53 −/− colon cancer cells were incubated with different concentrations (100–1000 mM) of MSM for 24 h before performing 3-(4,5-Dimethylthiazol-2-yl)-2,5 diphenyltetrazolium bromide (MTT) assay. Viability of cells incubated without MSM was considered as 100% and the results showed that MSM treatment inhibited cell viability of HCT-116 p53 +/+ cells between 200 and 1000 mM concentrations and HCT-116 p53 −/− cells between 100 and 1000 mM concentrations, dose-dependently and significantly (*p* < 0.05) (Figure 1).

### 2.2. MSM Induced Apoptosis of HCT-116 p53 +/+ and HCT-116 p53 −/− Colon Cancer Cells

**Figure 2:**
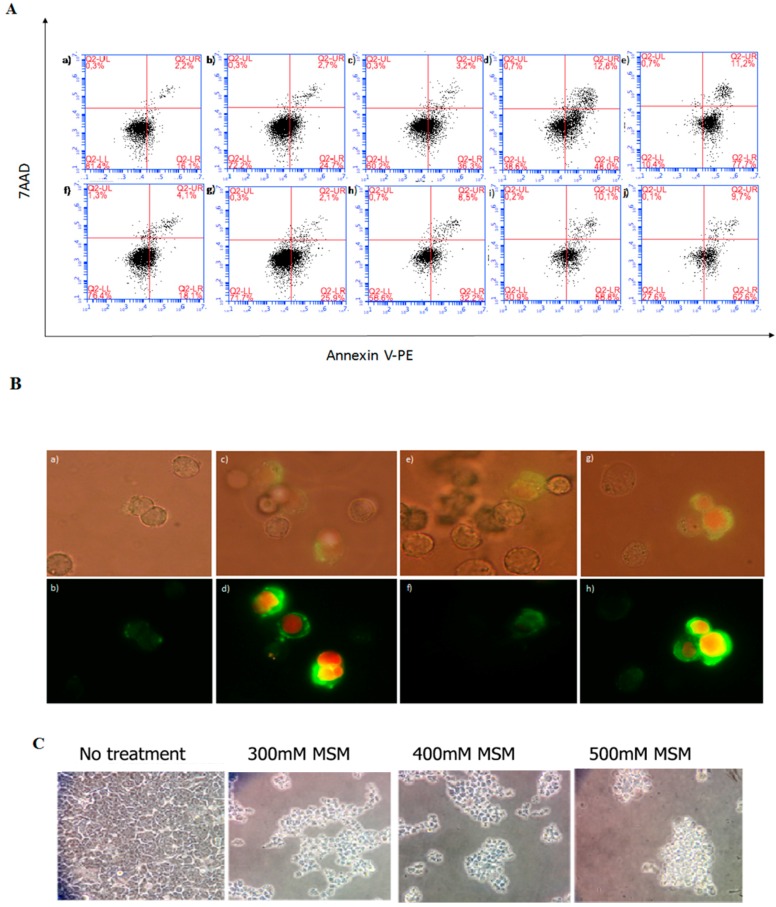

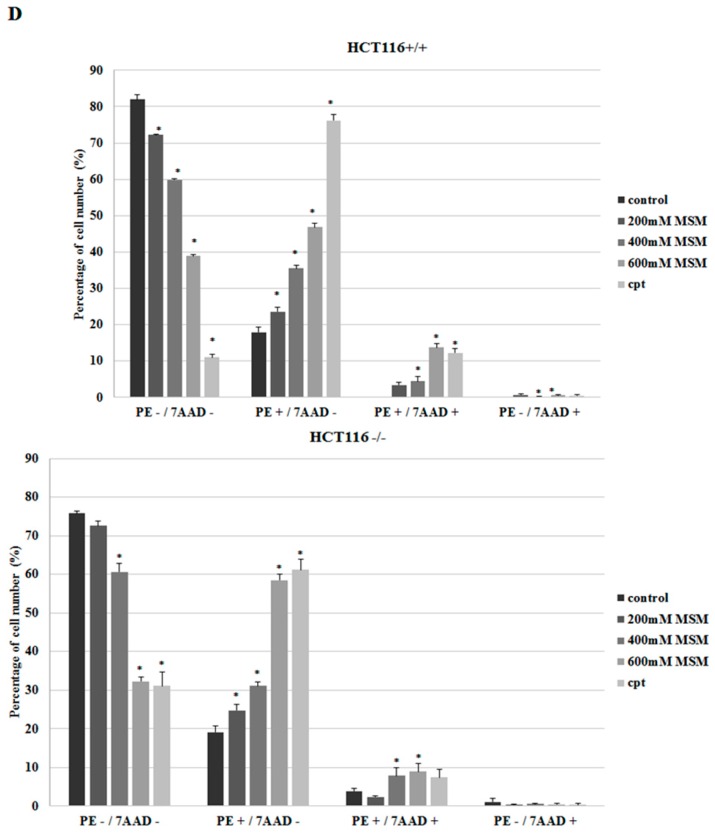
(**A**) Flow cytometric analysis of Annexin V-PE/7-AAD-stained HCT-116 p53 +/+ and p53 −/− colon cancer cells. HCT-116 colon cancer cells were incubated with MSM for 24 h before analyzing apoptosis with Annexin V-PE/7-AAD staining. Flow cytometry results are represented as (**a**) HCT-116 p53 +/+ cells without MSM; (**b**) HCT-116 p53 +/+ cells incubated with 200 mM MSM; (**c**) HCT-116 p53 +/+ cells incubated with 400 mM MSM; (**d**) HCT-116 p53 +/+ cells incubated with 600 mM MSM; (**e**) HCT-116 p53 +/+ cells incubated with camptothecin as a positive control; (**f**) HCT-116 p53 −/− cells without MSM; (**g**) HCT-116 p53 −/− cells incubated with 200 mM MSM; (**h**) HCT-116 p53 −/− cells incubated with 400 mM MSM; (**i**) HCT-116 p53 −/− cells incubated with 600 mM MSM; and (**j**) HCT-116 p53 −/− cells incubated with cpt as a positive control; (**B**) fluorescence microscopic examination (magnification 10 × 100) of Annexin V-FITC/PI-stained HCT-116 p53 +/+ and p53 −/− colon cancer cells. Annexin V-FITC (green) and PI (red) stained cells were incubated with or without MSM (400 mM) for 24 h. These constructs were examined by fluorescent microscopy under fluorescent and white light and fluorescent images were merged. (**a**,**b**) HCT-116 p53 +/+ cells (control); (**c**,**d**) HCT-116 p53 +/+ cells were incubated with 400 mM MSM; (**e**,**f**) HCT-116 p53 −/− cells (control); (**g**,**h**) HCT-116 p53 −/− cells were incubated with 400 mM MSM; (**C**) light microscopic examination (magnification 10 × 40) of HCT-116 p53 +/+ colon cancer cells incubated with or without MSM (300–500 mM) for 24 h; (**D**) flow cytometry results of HCT-116 p53 +/+ and p53 −/− colon cancer cells were shown as bar graphs. Percentage of viable cells (Annexin V-PE −/7-AAD−), early apoptotic cells (Annexin V-PE +/7-AAD−), late apoptotic cells (Annexin V-PE+/7-AAD+), and necrotic cells (Annexin V-PE −/7-AAD+) are shown as means ± SD. * *p* < 0.05 versus control (cells without MSM treatments).

In order to analyze the mode of cell death induced by MSM treatment, HCT-116 p53 +/+ and HCT-116 p53 −/− colon cancer cells were incubated with MSM (200, 400, and 600 mM) for 24 h before double-staining with Annexin V-PE/7-AAD. The results showed that all tested concentrations of MSM increased the number of early apoptotic (PE+/7-AAD−) and late apoptotic/dead (PE+/7-AAD+) HCT-116 p53 +/+ cells. MSM treatment also decreased the number of viable (PE−/7-AAD−) HCT-116 p53 +/+ cells, dose-dependently and significantly (*p* < 0.05) (Figure 2A,D). All tested concentrations of MSM also increased the number of early apoptotic (PE+/7-AAD−) HCT-116 p53 −/− cells (*p* < 0.05) (Figure 2A,D).

Fluorescence microscopic examinations after Annexin V-FITC/PI double-fluorescence staining also confirmed that MSM treatment caused apoptotic morphology in HCT-116 p53 +/+ and HCT-116 p53 −/− colon cancer cells (Figure 2B). Light microscopic examinations showed that MSM-treated HCT-116 p53 +/+ colon cancer cells lost their attachment (Figure 2C).

### 2.3. MSM-Increased Caspase-3 Activity in HCT-116 p53 +/+ and p53 −/− Colon Cancer Cells and Caspase-3 Inhibitor (Z-VAD-fmk) Rescued HCT-116 p53 +/+ and p53 −/− Colon Cancer Cells from MSM-Induced Cell Death

**Figure 3:**
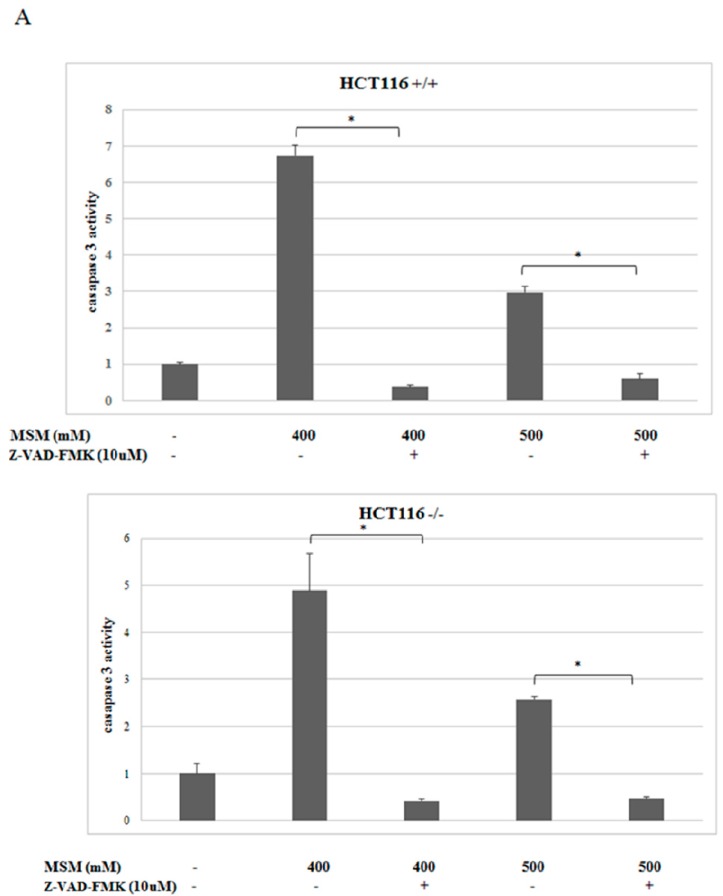

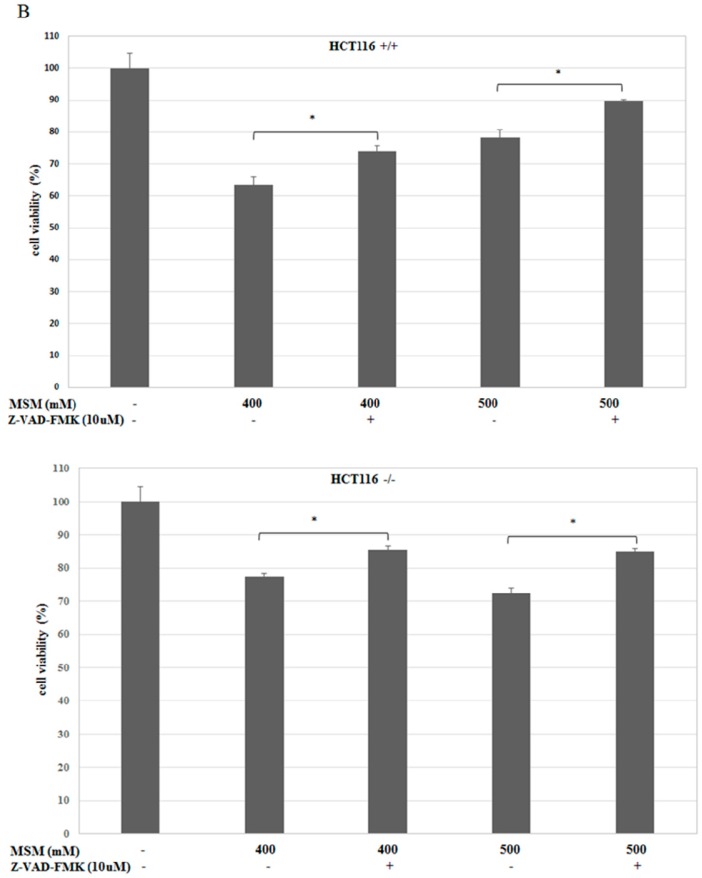
Effects of MSM on caspase-3 activity in HCT-116 p53 +/+ and p53 −/− colon cancer cells. Cells were treated with or without MSM (400 and 500 mM) for 24 h and cytosolic extracts of cells were used for caspase-3 activity. (**A**) Caspase-3 enzyme activity increased significantly after MSM treatment. The presense of Z-VAD-fmk (caspase-3 inhibitor) before 400 mM and 500 mM MSM treament decreased caspase-3 activity in both HCT-116 p53 +/+ and p53 −/− colon cancer cells; (**B**) cells were incubated with MSM (400 and 500 mM) in the absence and presence of Z-VAD-fmk. Cell viability of HCT-116 p53 +/+ and p53 −/− colon cancer cells increased in the presence of Z-VAD-fmk (* denotes a significant difference from the control group, *p* < 0.05).

The effect of MSM on caspase-3 enzyme activity was analyzed spectrophotometrically. It was found that MSM treatment (400 and 500 mM) significantly (*p* < 0.05) increased caspase-3 enzyme activity in HCT-116 p53 +/+ and HCT-116 p53 −/− colon cancer cells (Figure 3A). However, 400 mM MSM treatment resulted with a higher caspase-3 activity than 500 mM MSM treatment in both HCT-116 p53 +/+ and HCT-116 p53 −/− colon cancer cells. Furthermore, inhibition of caspase-3 activity by the use of caspase-3 inhibitor Z-VAD-fmk caused a significant (*p* < 0.05) increase in cell viability of both HCT-116 p53 +/+ and HCT-116 p53 −/− colon cancer cells, which suggest the involvement of caspase-3 in MSM-induced apoptosis.

### 2.4. MSM Induced c-Jun N-Terminal Kinases (JNK) Phosphorylation in Both HCT-116 p53 +/+ and p53 −/− Colon Cancer Cells

**Figure 4:**
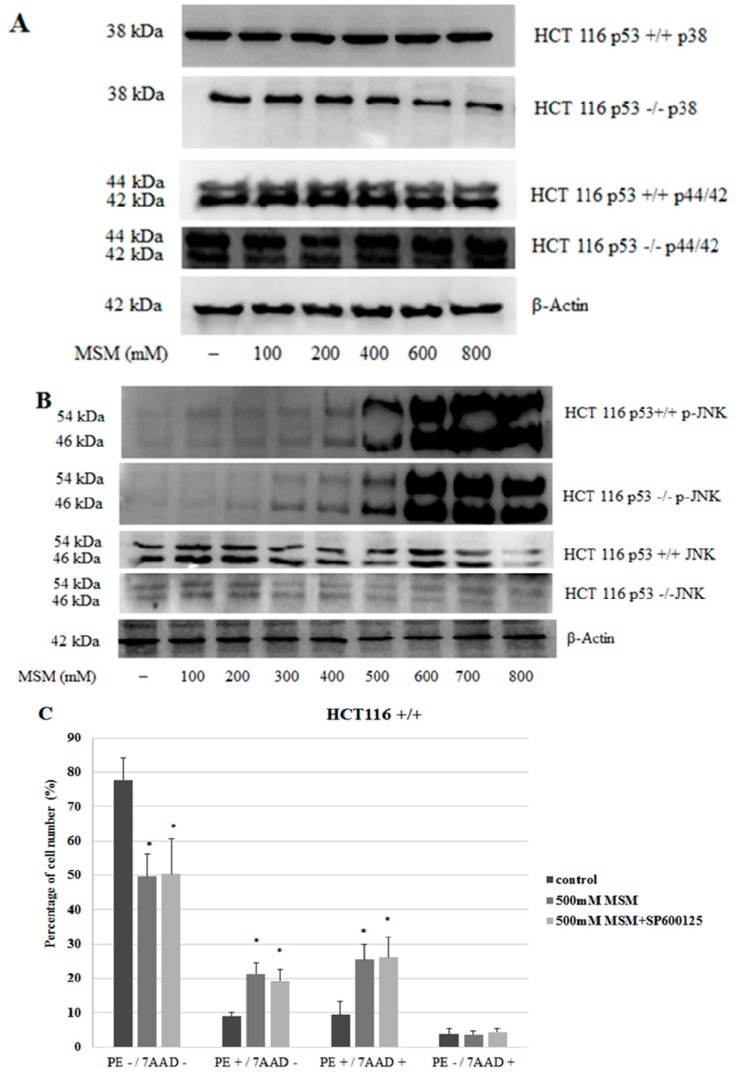

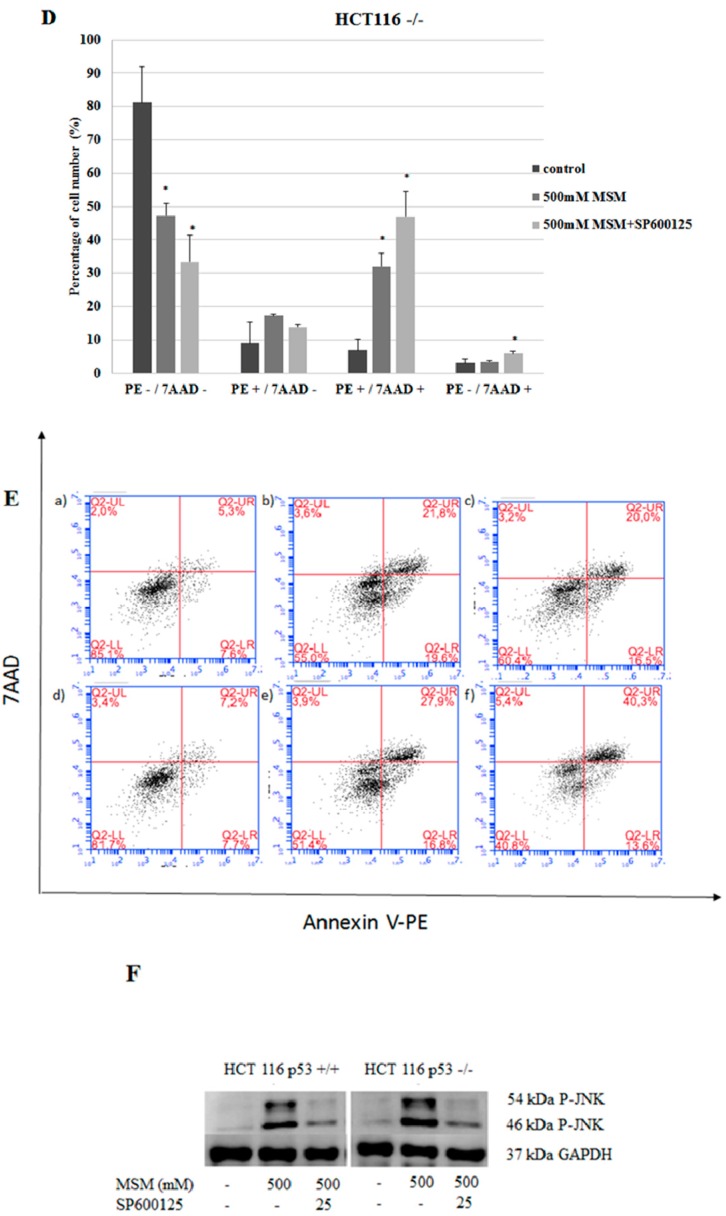

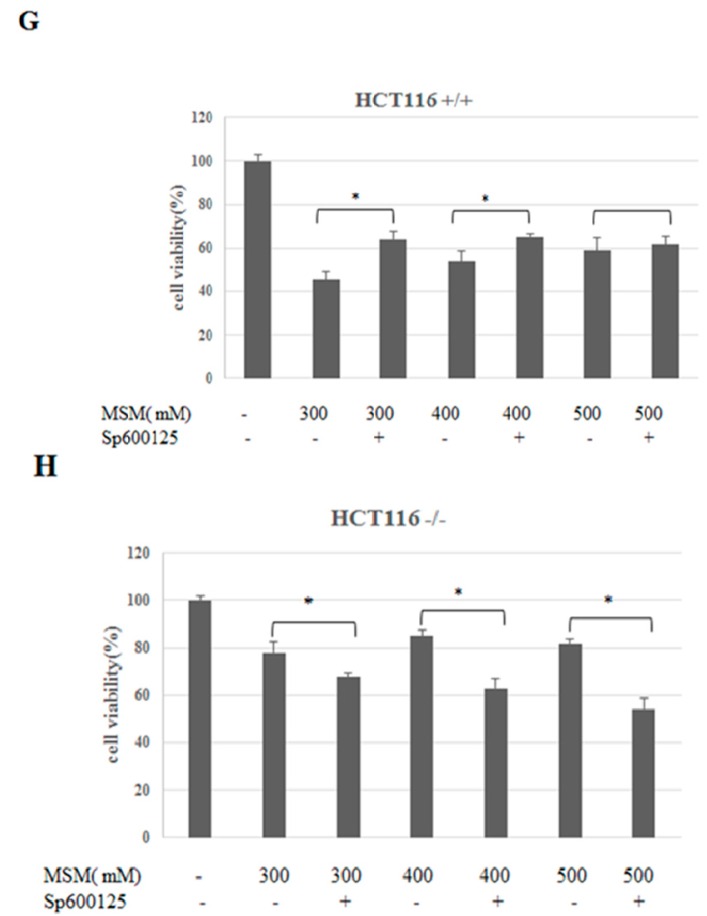
Effects of MSM on phosphorylated c-Jun N-terminal kinases (JNK) and unphosphorylated p38, p44/42, and JNK in HCT-116 p53 +/+ and p53 −/− colon cancers with or without MSM treatment. Cells were treated with MSM (100–800 mM) for 24 h and cell extracts were used for protein isolation and Western blotting for p38, p44/42, and JNK and p-JNK; β-actin was used as loading control. (**A**) Representative blot image showing the effect of MSM on unphosphorylated forms of p38 and p44/42 in HCT-116 p53 +/+ and p53 −/− colon cancer cells. No signal was detected for phosphorylated forms of p38 and p44/42 in HCT-116 p53 +/+ and p53 −/− colon cancer cells; (**B**) representative blot image showing the effect of MSM on unphosphorylated and phosphorylated forms of JNK. A very weak signal was detected for p-JNK in cells without MSM treatment. MSM treatment did not show any effect on unphosphorylated forms of p44/42, JNK, and p38 mitogen activated protein kinases (MAPKs) but HCT-116 p53 +/+ and p53 −/− colon cancer cells showed a significant increase in phosphorylated JNK at 200–800 mM; (**C**) bar graph representation of flow cytometry results in HCT-116 p53 +/+ cells; (**D**) bar graph representation of flow cytometry results in HCT-116 p53 −/− cells. In all experiments, freshly-prepared MSM in RPMI 1640 culture medium was used. Cells without MSM treatment were also maintained in the same RPMI 1640 culture medium; (**E**) HCT-116 p53 +/+ and p53 −/− colon cancer cells were incubated with 500 mM MSM in the presence and absence of 25 μM sp600125 (JNK inhibitor). Flow cytometry results are represented as (**a**) HCT-116 p53 +/+ cells without MSM and SP600125; (**b**) HCT-116 p53 +/+ cells incubated with 500 mM MSM; (**c**) HCT-116 p53 +/+ cells incubated with 500 mM MSM and SP600125; (**d**) HCT-116 p53 −/− cells without MSM and SP600125; (**e**) HCT-116 p53 −/− cells incubated with 500 mM MSM; and (**f**) HCT-116 p53 −/− cells incubated with 500 mM MSM and SP600125; (**F**) effect of JNK inhibitor SP600125 (SP) on MSM-induced JNK phosphorylation. HCT-116 p53 +/+ and p53 −/− colon cancer cells were incubated with 500 mM MSM in the presence and absence of 25 μM SP600125 (JNK inhibitor) and Western blot analysis was performed; (**G**) effect of JNK inhibitor SP600125 (SP) on MSM-induced cell viability loss in HCT-116 p53 +/+ colon cancer cells. Cells were incubated with MSM (300–500 mM) and SP600125 (25 μM), and cell viability was analyzed with MTT assay; (**H**) effect of JNK inhibitor SP600125 (SP) on MSM-induced cell viability loss in HCT-116 p53 −/− colon cancer cells. Cells were incubated with MSM (300–500 mM) and SP600125 (25 μM), and cell viability was analyzed with MTT assay (* *p* < 0.05 versus control group).

To clarify the mechanisms underlying the effects of MSM on cell death, MAPK proteins were also analyzed. Since MAPK proteins need to be phosphorylated for their activation, the effect of MSM treatment on phosphorylated and unphosphorylated forms of MAPKs were examined. In HCT-116 p53 +/+ and −/− cells, levels of unphosphorylated forms of p38 and p44/42 did not change significantly between cells with or without MSM treatment (Figure 4A). Our results showed that HCT-116 colon cancer cells without MSM treatment did not exhibit detectable amounts of p-JNK protein and MSM treatment induced the phosphorylation of JNK protein at 200–800 mM concentrations, dose-dependently (*p* < 0.05), in both HCT-116 p53 +/+ and −/− cells. P-JNK levels peaked with 600–800 mM concentrations of MSM in both HCT-116 p53 +/+ and −/− cells. Phosphorylated forms of p38 and p44/42 could not be detected in cell lysates of HCT-116 p53 −/− and HCT-116 p53 +/+ cells with or without MSM treatment. These results indicated that MSM mainly, and exclusively, induced phosphorylation of JNK among three MAPKs (Figure 4B) and the effects of MSM on cell viability status of HCT-116 colon cancer cells might have been mediated by its effects on the JNK pathway. To test the involvement of JNK phosphorylation in MSM-induced apoptosis, HCT-116 p53 +/+ and HCT-116 p53 −/− colon cancer cells were pre-incubated with JNK inhibitor SP600125 (25 μM) for one hour before incubation with MSM (500 mM) for 24 h. Our MTT results showed that JNK inhibitor SP600125 administration before MSM (500 mM) treatment significantly improved HCT-116 p53 +/+ cell viability in comparison to HCT-116 p53 +/+ cells treated with only MSM (500 mM), whereas it potentiated the loss in HCT-116 p53 −/− cell viability (Figure 4G–H). To analyze the effect of SP600125 on apoptosis, cells were stained with Annexin V-PE/7-AAD and analyzed with flow cytometry. Our results showed that SP600125 pre-treatment before MSM treatment caused a decrease in the number of early apoptotic (PE+/7-AAD−) HCT-116 p53 +/+ and HCT-116 p53 −/− colon cancer cells. However, these changes were not found as statistically significant (Figure 4C–E). SP600125 pre-treatment before MSM treatment increased the number of late apoptotic HCT-116 p53 −/− colon cancer cells (Figure 4D,E).

### 2.5. MSM Treatment Modulated the Expressions of Various Genes and Proteins Involved in Apoptosis

**Figure 5:**
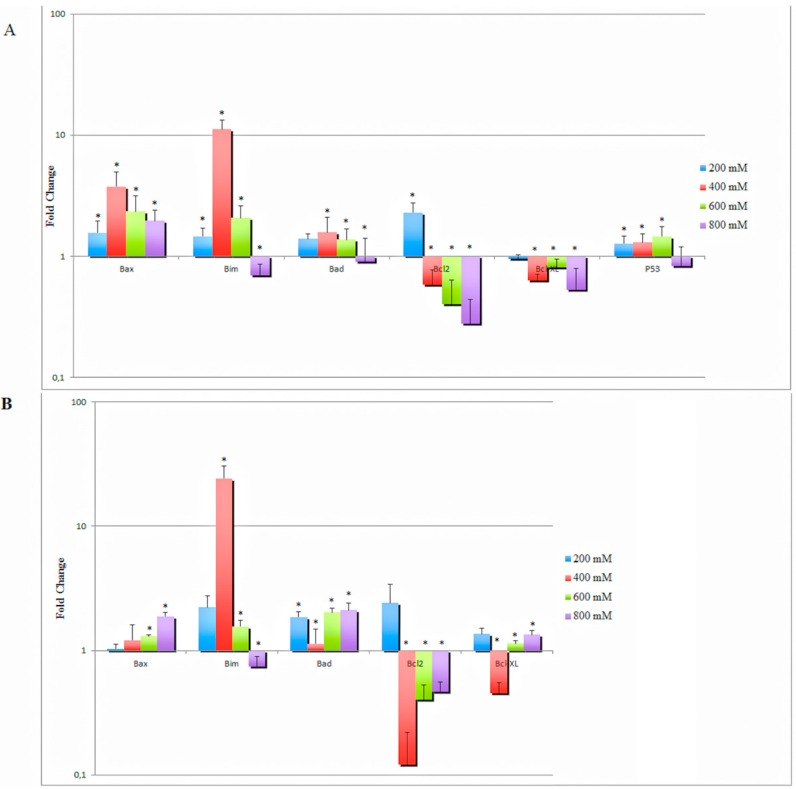
Effects of MSM treatment on pro-apoptotic and anti-apoptotic gene expressions in (**A**) HCT-116 p53 +/+ and (**B**) p53 −/− colon cancer cells. Pro-apoptotic and anti-apoptotic gene expressions were measured using quantitative RT-PCR. After 24 h incubation of cells with MSM (200–800 mM), total RNA was extracted, cDNA was synthesized, and quantitative RT-PCR was performed. Hypoxanthine-guanine phosphoribosyltransferase (*HPRT*) was used as an endogenous control. MSM (200–800 mM) treatment modulated the expressions of pro-apoptotic genes *Bax*, *Bim*, and *Bad*, and anti-apoptotic genes *Bcl-2* and *Bcl-xL* (* denotes a significant difference from the control group, *p* < 0.05).

The in vitro effect of MSM on specific apoptosis-related genes was also analyzed. All concentrations of MSM increased *Bax* gene expression in both HCT-116 p53 +/+ and HCT-116 p53 −/− cells, whereas 200–600 mM MSM treatment of HCT-116 p53 +/+ and HCT-116 p53 −/− cells resulted with increased pro-apoptotic *Bim* gene expression levels (*p* < 0.05). MSM treatment also increased gene expression levels of *Bad* in both cell lines at all concentrations, except the decreased expression of *Bad* in HCT-116 p53 +/+ cells treated with 800 mM MSM. MSM treatment induced a similar gene expression profile of *Bcl-2* in both cell lines exhibiting increased *Bcl-2* expression in 200 mM MSM-treated cells and decreased *Bcl-2* expression in 400–800 mM MSM-treated cells. In summary, treatment of HCT-116 p53 −/− cells with 400 mM MSM resulted with the highest *Bax/Bcl-2* ratio among all concentrations. The *Bax/Bcl-2* ratio also increased in HCT-116 p53 +/+ cells more than six-fold with 400 mM MSM treatment. All concentrations of MSM decreased *Bcl*-*xL* expression in HCT-116 p53 +/+ cells, whereas only 400 mM MSM treatment caused decreased *Bcl-xL* expression in HCT-116 p53 −/− cells. MSM (200–600 mM) treated HCT-116 p53 +/+ cells displayed increased *p53* gene expression (*p* < 0.05) (Figure 5A).

It can be concluded that the effects of MSM on the expression of apoptotic family genes varied between its different concentrations. The most dramatic change was seen in *Bim* gene expression, which increased more than 10-fold in both HCT-116 p53 +/+ and p53 −/− cells after 400 mM MSM treatment. For most of the genes, 400 mM MSM treatment induced a stronger effect in favor of apoptosis than >400 mM MSM treatment.

**Figure 6:**
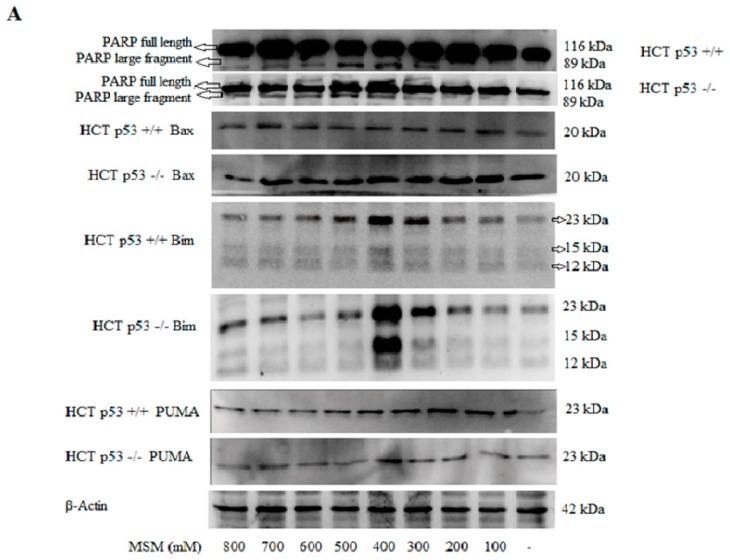

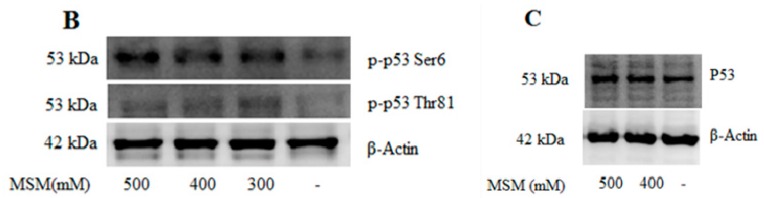
Cells were treated with MSM (100–800 mM) for 24 h and cell extracts were used for protein isolation and Western blotting; β-actin was used as loading control. (**A**) Representative blot image showing the effects of MSM on poly (ADP-ribose) polymerase (PARP) cleavage, Bax, Bim, and p53 upregulated modulator of apoptosis (PUMA) proteins in HCT-116 p53 +/+ and p53 −/− colon cancer cells; (**B**) representative blot image showing the effects of MSM on p-p53 (Thr81) and p-p53 (Ser6) proteins in HCT-116 p53 +/+ colon cancer cells. Representative blot image showing the effect of MSM on p-p53 (Thr81) and p-p53 (Ser6) in HCT-116 p53 +/+ colon cancer cells; (**C**) representative blot image showing the effect of MSM (400 and 500 mM) on p53 protein in HCT-116 p53 +/+ colon cancer cells.

In addition to our RT-PCR results, Western blot analysis demonstrated that all concentrations of MSM, except 800 mM, increased Bax (Figure 6A) protein expression. The most dramatic effect of MSM was again seen on Bim protein expression, which gradually and dose-dependently increased with 200–400 mM MSM treatment. Bim was expressed at its highest level with 400 mM MSM treatment in both HCT-116 p53 +/+ and −/− cells. However, Bim expression began to decrease at concentrations >400 mM MSM. PUMA protein levels also displayed a similar pattern to Bim and increased with 200–400 mM concentrations of MSM.

To investigate whether JNK phosphorylation led to p53 phosphorylation and subsequent p53 accumulation, western blot analysis was performed on HCT-116 p53 +/+ and −/− cell extracts treated with MSM. Western blot analysis showed that MSM treatment of HCT-116 p53 +/+ cells significantly induced p53 Thr81 and Ser6 phosphorylation (Figure 6B) and p53 accumulation (Figure 6C). These data suggest that modulation of cell viability by MSM may involve the activation of the p53 pathway and subsequent expression of apoptotic family genes which are downstream targets of p-JNK and p-p53.

Cells were also analyzed for PARP fragmentation and blotted for full length and cleaved PARP. 300–800 mM concentrations of MSM increased protein levels of 89 kDa PARP cleavage fragments significantly, which was dose-dependent between 300 and 500 mM (*p* < 0.05) in both HCT-116 p53 +/+ and p53 −/− cells (Figure 6A). However, even though PARP fragmentation increased in 300–800 mM MSM-treated cells in comparison to untreated cells, protein levels of 89 kDa PARP fragments decreased with concentrations of MSM higher than 500 mM in comparison with 300–500 mM MSM-treated cells. The effect of MSM on PARP fragmentation did not differ between HCT-116 p53 +/+ and p53 −/− cells and, therefore, occurred independently of p53.

## 3. Discussion

This study was designed to disclose the mechanisms underlying apoptosis induced by MSM in HCT-116 p53 +/+ and HCT-116 p53 −/− colon cancer cells. For this purpose, firstly the effects of MSM on cell proliferation and apoptosis were examined with a colorimetric MTT cell viability test and Annexin V-PE/7-AAD flow cytometric analysis. It was found that MSM treatment inhibited cell viability and induced apoptosis of HCT-116 p53 +/+ and HCT-116 p53 −/− colon cancer cells. The increase in PARP fragmentation and caspase-3 activity in both cell lines after treatment with MSM also supported the flow cytometry data. Prior studies have similarly reported MSM-induced apoptosis in AGS, HepG2, and KYSE-30 colon cancer cells at 28.04 mg/mL (297.88 mM), 21.87 mg/mL (232 mM), and 27.98 mg/mL (297.24 mM) concentrations, respectively [34]. MSM has also been reported to activate PARP cleavage and apoptosis in HepG2, Huh7-Mock, and Huh7-H-rasG12V liver cancer cell lines at 500 mM concentration [8]. According to our data, inhibition of caspase-3 activity with Z-VAD-fmk reversed the decline in cell viability induced by MSM in both cell lines. This suggests that caspase-3 activation is involved in MSM-induced apoptosis. 

To further investigate apoptosis induced by MSM, we analyzed MAPK proteins which play essential roles in the regulation of signaling events related to cell viability and apoptosis. Our results showed that MSM treatment induced the phosphorylated form of JNK, whereas it did not exert any effect on the phosphorylated forms of p44/42 and p38 MAPKs. Disturbances in JNK regulation have been reported to show correlation with cancer [25,26]. It has been postulated that JNK functions as an anti-apoptotic protein in cancer cells and contributes to tumorigenesis [35,36]. However, numerous studies have indicated that JNK may also trigger apoptosis [33,37] after its activation through a phosphorylation mechanism [27,28,29]. Our results indicated that MSM treatment induced JNK phosphorylation in a dose-dependent manner. JNK inhibitor SP600125 (SP) significantly reversed the decrease in HCT-116 p53 +/+ cell viability induced by MSM. Inhibition of p-JNK with SP also decreased the number of early apoptotic HCT-116 p53 +/+ cells without statistical significance. These results suggest that JNK activation may, partly, have an effect on the loss of cell viability induced by MSM in HCT-116 p53 +/+ cells. It has been reported that one of the pathways leading to JNK-mediated apoptosis involves the phosphorylation of p53 [38] at Ser6 and/or Thr81 and this phosphorylation inhibits ubiquitin-mediated degradation of p53 and thereby stabilizes the levels of p53 [39,40]. p53 orchestrates various biological events, including cell cycle arrest, cellular senescence, and DNA repair, in addition to apoptosis. Hence, our data showing the increase in cell viability after SP treatment may depend on the inhibition of phosphorylation and subsequent accumulation of p53 downstream of p-JNK. On the other hand, inhibition of p-JNK did not increase cell viability in MSM-treated HCT-116 p53 −/− cells. This suggests that JNK activation is not essentially required for the induction of apoptosis.

Since our data showed that MSM-induced apoptosis is p53-independent, its apoptotic effects on the modulation of *Bax* and *Bcl*-2 did not require p53. A high *Bax*/*Bcl-2* ratio was achieved in both cell lines after 400 mM MSM treatment. Recently, it was reported that MSM induced Bax protein expression in both MCF-7 and T47D breast cancer cell lines [7], and decreased Bcl-2 protein expression and suppressed hepatic tumor development through the activation of apoptosis [8]. On the other hand, in our previous study we showed that MSM inhibited nitric oxide induced RAW 264.7 macrophage apoptosis at its moderately lower concentrations (50 mM) by decreasing Bax/Bcl-2 ratio, whereas it increased apoptosis at high concentrations (200 mM) [41]. In another study MSM was found to protect liver cells against CCl4 induced injury and apoptosis by reducing Bax/Bcl-2 ratio [6]. Collectively, all of this data presents that MSM can act as an inducer or suppressor of Bax and Bcl-2 depending on its concentration or the biological model that is used.

It has been shown that, following upregulation of *Bax* gene expression, executioner caspase-mediated PARP cleavage promotes apoptosis [42]. We also found increased caspase-3 activity and PARP fragmentation which might be induced after the upregulation of the *Bax* gene.

Even though all concentrations of MSM induced PARP fragmentation in comparison with untreated cells, the most potent increase in PARP fragmentation was seen with 400 and 500 mM MSM treatment in both cell lines. Similar to the expression changes of most other apoptotic genes and proteins, it was seen that this increase began to decline after 500 mM MSM treatment, but was still significantly higher than untreated cells.

One of the most striking findings in this study is the dramatic increase in *Bim* gene and protein expression in HCT-116 p53 +/+ and HCT-116 p53 −/− colon cancer cells treated with 400 mM MSM. Bim is a pro-apoptotic Bcl-2 homology 3-only protein which was first discovered by O’Connor et al. [43]. In humans, three isoforms of Bim have been identified: BimEL, BimL, and Bim S. All three isoforms of Bim have been reported to induce apoptosis [43]. In recent years Bim has been gaining serious attention for the development of chemotherapeutical agents because of its critical roles in tumorigenesis, metastasis, and chemoresistance. It has been reported that Bim expression decreases in carcinogenesis and tumor cells develop different mechanisms to suppress Bim expression to resist apoptosis, which otherwise results with their elimination. Various anti-cancer drugs depend on Bim to be effective, whereas deficiencies in Bim induction may result in therapy failure [44]. It has also been suggested that Bim-targeting therapies may provide more effective and unique concepts for the management and treatment of cancer [45]. Overall, because of the strong inducing effect of MSM (400 mM) on Bim expression, MSM has the potential to be an ideal candidate drug for future cancer therapies. Moreover, Bim has a phosphorylation site for JNK, which displays their possible interaction. However, the role of JNK for Bim is still unclear. Phosphorylation of Bim by JNK promotes proteasomal Bim degradation in the T cell acute lymphoblastic leukemia cell line Sup-T [46]. However, various studies have suggested the pro-apoptotic activity of JNK-mediated Bim phosphorylation, which decreases the binding of Bim to the antiapoptotic protein Bcl-2, and activates Bim as a result [47]. We found that JNK was phosphorylated in 400 mM MSM-treated cells where Bim was also strongly activated. However even JNK phosphorylation continued to increase gradually with increasing concentrations of MSM and peaked at 600–800 mM, Bim expression began to decline with >400 mM MSM. Therefore, a strong Bim activation was not seen with MSM concentrations which induced the highest p-JNK levels. Further research is needed to discover the interaction of Bim and p-JNK in MSM-induced apoptosis.

In conclusion, our results show for the first time that MSM induces apoptosis in HCT-116 colon cancer cells regardless of their p53 status. Since over 50% of human tumors contain a functionally defective p53 that diminishes sensitivity to commonly used chemotherapeutic agents, the ability of MSM to induce apoptosis independently of p53 may offer an advantage in anti-tumor therapy. Moreover, the remarkable effect of MSM on Bim also suggests its potential use as a novel chemotherapeutic agent for Bim-targeted anti-cancer therapies.

## 4. Materials and Methods

### 4.1. Chemicals

McCoy’s 5A medium, fetal bovine serum (FBS), penicillin/streptomycin, and l-glutamine were from PAA (Pasching, Austria). 3-(4,5-Dimethylthiazol-2-yl)-2,5 diphenyltetrazolium bromide (MTT) and dimethylsulfoxide (DMSO) were from Sigma (St Louis, MO, USA). Annexin V-PE, 7-AAD, Annexin V-FITC, and PI were from BD Biosciences (San Diego, CA, USA). Caspase-3 activity assay kit was from Abcam (Cambridge, MA, USA). Bradford agent was from Bio-rad (Hemel Hempstead, UK). Bovine serum albumin (BSA) solution was from Thermo (Rockford, IL, USA). MSM was from Aldrich (St Louis, MO, USA). Phospho-MAPK Family Antibody Sampler Kit (Phospho-p38 MAPK Thr180/Tyr182, Phospho-p44/42 MAPK Thr202/Tyr204, Phospho-SAPK/JNK Thr183/Tyr185), MAPK Family Antibody Sampler Kit (p38 MAPK, p44/42 MAPK, SAPK/JNK), primary antibodies for PARP (rabbit polyclonal), p53 (1C12 mouse monoclonal), p-p53-Thr81 (rabbit polyclonal), p-p53-Ser6 (rabbit polyclonal), PUMA (rabbit monoclonal), Bim (rabbit monoclonal), Bax (rabbit polyclonal) were from Cell Signaling (Danvers, MA, USA) and used at 1:1000 dilution, JNK inhibitor (SP600125) was from Cell Signaling (Danvers, MA, USA), anti-β-actin antibody (AC-15 mouse monoclonal) was from Sigma and used at 0.5 mg/mL dilution. Secondary anti-rabbit Ig G-HRP and anti-mouse Ig G-HRP antibodies were from Cell Signaling (Danvers, MA, USA) and used at 1:1000 dilution. Loading buffer for Western blot was from New England Biolabs (Beverly, MA, USA). Z-VAD-fmk was from Tocris Biosciences (Bristol, UK), High Pure RNA kit, Transcriptor High Fidelity cDNA Synthesis Kit and SYBR Green PCR Master Mix were from Roche (Mannheim, Germany).

### 4.2. Cell Culture

HCT-116 p53+/+ and HCT-116 p53 −/− colon cancer cells were kindly provided by Bert Vogelstein and were cultured in 75 cm^2^ flasks, at 37 °C and 5% CO_2_ saturated air, in McCoy’s 5A medium containing 10% FBS, 1% l-glutamine, and 1% penicillin-streptomycin. FBS was inactivated in a 60 °C water bath for 30 min before medium preparation. Cells were trypsinized from flasks for passaging when they became 80% confluent. Cells were counted with trypan blue to seed an equal number of cells to wells.

### 4.3. Cell Viability Assay

MTT assay was used to determine the cytotoxic effects of MSM against HCT-116 colon cancer cells. The principle of the assay relies on the reduction of tetrazolium salt by mitochondrial dehydrogenase in viable cells [48]. Stock solutions of MSM were prepared in McCoy’s 5A culture medium. Briefly, cells were seeded (6 × 10^4^ cells/well) in a 96-well plate before treatment with different concentrations (100–1000 mM) of MSM. After 24 h incubation, MTT solution was added to each well at a final concentration of 0.5 mg/mL. After 2 h of incubation, the supernatants were aspirated before dissolving the formazan product with 100 μL dimethylsulfoxide (DMSO). The absorbance at 540 nm was then read using a spectrophotometric microplate reader. Viability of cells without treatment was considered as 100% and results were calculated as viability % in comparison to non-treated cells. For JNK inhibition and caspase-3 activity assays, cells were pre-treated with JNK inhibitor (SP600125) and Z-VAD-fmk, which were dissolved in DMSO and applied to the medium of cells at final concentrations of 20 and 10 µM, respectively. Final concentration of DMSO in the incubation medium never exceeded 0.3% and cells without treatment were incubated with the same concentration of DMSO. 

### 4.4. Apoptosis Analysis by Flow Cytometry

Apoptosis was measured by Annexin V-PE/7-AAD double-staining. Briefly, HCT-116 p53+/+ and HCT-116 p53 −/− colon cancer cells (8 × 10^5^ cells/well) were seeded in six-well plates. Following incubation of cells with MSM (300, 400, and 500 mM) for 24 h, MSM-treated cells and cells without treatment were trypsinized, collected, and double-washed with PBS (phosphate-buffered saline) before resuspending in 1× binding buffer (500 μL) according to manufacturer’s instructions (Annexin V-PE/7-AAD apoptosis detection Kit, BD Biosciences). 100 μL of cell suspension was incubated with 5 μL Annexin V-PE and 5 μL 7-AAD at room temperature for 15 min. Cells were briefly vortexed before incubation for 15 min in the dark at room temperature. After this incubation, 400 μL 1× binding buffer was added to cells before flow cytometry analysis by Accuri C6 flow cytometry. The amounts of early apoptosis and late apoptosis were determined as the percentage of Annexin V-PE +/7-AAD− or Annexin V-PE +/7-AAD + cells, respectively.

### 4.5. Light and Fluorescence Microscopy

After proper treatments, cells were visualized under a light microscope. Cells with or without MSM were also visualized under a fluorescent microscope after staining with Annexin V-FITC/PI.

### 4.6. Caspase-3 Activity Assay

Cells were lysed and lysates with equal amounts of protein were incubated with caspase-3 colorimetric DEVD-pNA substrate at 37 °C. The resulting colorimetric product was measured with a Multiskan™ GO microplate spectrophotometer (Thermo) at 402 nm according to the instructions of the Abcam Caspase-3 Kit, and caspase-3 activity was calculated as a fold change in comparison to cells without treatment.

### 4.7. Western Blotting Analysis

Western blotting was performed as previously described [41]. The cells were harvested, washed with PBS, and lysed with Nuclear Extract Kit according to the manufacturer’s instructions (Active Motif, Carlsbad, CA, USA). Cell lysates were mixed with NEB 3× reducing blue loading buffer for denaturation. Equal amounts of protein were loaded to sodium dodecyl sulfate-polyacrylamide gel electrophoresis (SDS-PAGE) gels before transfer on to PVDF membranes. Bio-Rad Tetra Cell Blotting module was used for protein transfer from gels to membranes. After 1 h of transfer, membranes were washed with PBS and then blocked in PBS containing 5% non-fat dried milk. Membranes were then incubated with specific primary antibodies overnight at 4 °C. After three washes in PBS-Tween, membranes were incubated with the appropriate HRP conjugated secondary antibodies for 1 h at room temperature. Membranes were then washed with PBS-tween before detection step using an enhanced chemiluminescence Western blotting detection (Super signal West Femto Signal Fire agent of Thermo). β-actin was used as a housekeeping protein and loading control to confirm equal loading of protein to gels. Band intensities were analyzed by Carestream MI software (Carestream Health Inc., Rochester, NY, USA). Final quantitations were made by dividing the band intensities of target proteins to the band intensities of housekeeping protein β-actin. Data of specific protein levels are presented as fold changes relative to the control. The results were subjected to analysis of variance (ANOVA) followed by the Student Newman Keuls test to analyze differences between conditions. In each case, a *p* value of <0.05 was considered to be statistically significant.

### 4.8. RNA Extraction, cDNA Synthesis, and Quantitative Real-Time Polymerase Chain Reaction (qPCR)

For detection of apoptosis-related gene expression levels, we used quantitative real-time PCR. HCT-116 colon cancer cells were seeded in six-well plates. Cells were incubated with different concentrations of MSM for 24 h. After the incubation period, total RNA was extracted from HCT-116 colon cancer cells by using the High Pure RNA kit (Roche, Mannheim, Germany) according to the manufacturer’s protocols. cDNA was generated from RNA by reverse transcriptase (Transcriptor High Fidelity cDNA Synthesis Kit; Roche). Quantitative Real-Time Polymerase Chain Reaction (qPCR) was performed using SYBR Green PCR Master Mix (Roche) on a LC480 instrument (Roche Diagnostics GmbH, Mannheim, Germany). mRNA was measured relative to hypoxanthine-guanine phosphoribosyltransferase (*HPRT*) as an endogenous control. The threshold cycle (*C*t) number was determined and used in the comparative Ct method. The relative quantity of the target gene was estimated by the 2^−ΔΔ*C*t^ method. Experiments were performed in biological triplicates. The primer sequences used were: *HPRT* forward: TGACACTGGCAAAACAATGCA; reverse: GGTCCTTTTCACCAGCAAGCT (product size: 94 bp); *Bad* forward: GATGAGTGACGAGTTTGTGGA; reverse: CAAGTTCCGATCCCACCAG (product size: 130 bp); *Bax* forward: GACGGCAACTTCAACTGGG; reverse: AGGAGTCTCACCCAACCAC (product size: 182 bp); *Bim* forward: ATCTCAGTGCAATGGCTTCC; reverse: CATAGTAAGCGTTAAACTCGTCTCC, (product size: 111 bp); *Bcl-2* forward: CGCCCTGTGGATGACTGAGT; reverse: GGGCCGTACAGTTCCACAA (product size: 93 bp); *p53* forward: CTTTCCACGACGGTGACA; reverse: TCCTCCATGGCAGTGACC (product size: 70 bp) (Roche); *Bcl-xL* forward: GATCCCATGGCAGCAGTAAAGCAAG; reverse: CCCCATCCCGGAAGAGTTCATTCACT (product size: 164 bp) [49]. 

### 4.9. Statistical Analysis

Statistical analysis was performed with a one-way ANOVA variance analysis test with post-hoc Student-Newman-Keuls test by using the StatistiXL program (Broadway–Nedlands, Western Australia). *p* < 0.05 was considered as significant. Results were expressed as mean ± standard deviation. One-sample *t*-test was used for RT-PCR analysis.
